# A PZT Actuated Triple-Finger Gripper for Multi-Target Micromanipulation

**DOI:** 10.3390/mi8020033

**Published:** 2017-01-24

**Authors:** Tao Chen, Yaqiong Wang, Zhan Yang, Huicong Liu, Jinyong Liu, Lining Sun

**Affiliations:** Jiangsu Provincial Key Laboratory of Advanced Robotics & Collaborative Innovation Center of Suzhou Nano Science and Technology, Soochow University, Suzhou 215123, China; chent@suda.edu.cn (T.C.); 20154229003@stu.suda.edu.cn (Y.W.); yangzhan@suda.edu.cn (Z.Y.); 20134229002@stu.suda.edu.cn (J.L.); lnsun@hit.edu.cn (L.S.)

**Keywords:** micromanipulation, triple-finger, micro-gripper

## Abstract

This paper presents a triple-finger gripper driven by a piezoceramic (PZT) transducer for multi-target micromanipulation. The gripper consists of three fingers assembled on adjustable pedestals with flexible hinges for a large adjustable range. Each finger has a PZT actuator, an amplifying structure, and a changeable end effector. The moving trajectories of single and double fingers were calculated and finite element analyses were performed to verify the reliability of the structures. In the gripping experiment, various end effectors of the fingers such as tungsten probes and fibers were tested, and different micro-objects such as glass hollow spheres and iron spheres with diameters ranging from 10 to 800 μm were picked and released. The output resolution is 145 nm/V, and the driven displacement range of the gripper is 43.4 μm. The PZT actuated triple-finger gripper has superior adaptability, high efficiency, and a low cost.

## 1. Introduction

Applications of micro-manipulation can be extensively found in microsystem manufacture, micro-medical, biomedical, optical engineering, and other important areas [1]. An increased requirement in micro-manipulation has led to rapid development of manipulating grippers. For micro/nano-manipulation, the manipulated objects usually have a small size, typically ranging from 1 μm to 1 mm, and are difficult to handle. Hence, various micro-grippers have been designed and developed based on different actuation techniques.

Grippers in the form of double- or triple-finger are normally driven by magnetic, electrostatic, or piezoelectric actuation mechanisms [2]. The implementation of magnetic actuator requires a strict manipulating environment, which cannot be affected by the ambient magnetic field interaction. Due to the disadvantageous scaling of magnetic fields, magnetic microactuators have low force output which limited their performance. The electrostatic actuation mechanism based on micro-electromechanical systems (MEMS) can realize high precision with a small device size and generate a satisfactory amount of output force [3,4]. But, the integrated structure is fragile and the micro-fabrication process is complicated. Xu et al. developed a micro-gripper with integrated electrostatic actuator and capacitive force sensor. The feasibility and effectiveness of the gripper device were validated by gripping a human hair 76 μm of diameter [5]. Boudaoud et al. fabricated an electrostatic micro-gripper with a nonlinear actuation mechanism while handling calibrated micro glass balls 80 μm of diameter [6]. Brandon et al. made a MEMS gripper which can pick and place gold nano spheres. The gripper was able to withstand gripping forces more than 700 μN and 38 μm out-of-plane bending deflections, proving the gripping tips to be mechanically strong for micro/nano manipulation [7]. The pick-and-place of a 100 nm gold nano sphere inside an SEM has been demonstrated with this gripper. Due to limitation of micro-fabrication process, the manipulation range of these two electrostatic grippers cannot be adjusted. Piezoelectric micro-actuators, with their high speed and good motion resolution, have been widely advocated to implement high precision grasping manipulation [8]. A major disadvantage of a piezoelectric actuation mechanism is that the output motion is relatively small (typically about 10–20 μm). To solve this problem, displacement amplification mechanisms are usually employed [9]. Wang et al. developed a monolithic compliant piezoelectric-driven micro-gripper with a large displacement amplification ratio of 16 [10].

Grippers with a wide clamping range are adaptable to manipulate various objects. Thus, the manipulation efficiency can be greatly improved. Sun et al. developed a piezo-actuated flexure-based micro-gripper being capable of handling various sized micro-objects with a maximum jaw displacement of 150.8 μm and a high amplification ratio of 16.4 [11]. It adopted monolithic structure and had a fixed manipulating range. Nevertheless, most structures of the reported micro-grippers cannot be changed according to different targets, thus confining the jaw displacement to variously sized micro-objects. Compared with the monolithic structure of a micro-gripper, a multi-actuator system driven by numerous identical single actuators connected in parallel and in series is highly adaptable to handle different objects. In this light, it is the best choice for the mechanical structure design of the micro-gripper.

A common design attribute of the micro-grippers proposed in recent years is a double-finger design [12], which has been proved to have a better performance in grasping micro-objects [13,14]. However, it could not release objects effectively due to the adhesion force [15]. In the micro scale, the strong adhesion force would make the micro-object adhere to the end-effector during release, especially for the objects under 50 μm. To solve this issue, many researchers have conducted significant work on the approaches to ease the difficulty of release. Those solutions and manipulation strategies can be roughly divided into two types, passive release and active release.

The basic idea of passive release is to increase the adhesion force between object and substrate or decrease the adhesion force between object and gripper. Liquid with great viscosity was painted on the basement to increase the adhesion force between object and substrate [16]. By scrolling the object, contacting surface can be reduced to achieve a success release [17]. These strategies are strongly dependent on the surface properties of substrate, and are greatly influenced by the materials and environment. Each process of release would take a very long time with low efficiency and poor operation repeatability.

Other approaches for the separation of objects and manipulators are proposed by using an external electric field, magnetic field force [18] or liquid frozen method [19], which called active release without contacting with the substrate. Those kinds of releasing ways do not depend on the surface properties of the substrate, but have difficulty in the miniaturization of grippers and require complex peripherals. Yet the release precision is poor. Sun et al. presented a novel MEMS gripper with a plunging structure for releasing the objects adhered to a gripping arm [20]. Although these triple-arm grippers are able to releasing the microspheres, the temporary impact of the plunging arm is so strong that the structure of some soft micro-objects may be damaged. And, the tilt thrust may affect the release accuracy to a certain extent. High frequency mechanical vibration of manipulation tools for object releasing are also conducted though piezoelectric ceramics driving [21], while the precise positioning is not achieved.

This paper reports the design of a separate-structured triple-finger gripper with a piezoelectric actuation mechanism for multi-target micromanipulation. Different from those grippers with gripping arms or fingers distributed in the same plan, this triple-finger gripper are designed in a spatial distributed structure enabling highly stable and repeatable pick-and-place of micro-objects. Grippers described above are limited in their clamping range. By adjusting the positions of the three separate assembly modules, which can be replaced by a variety of components suitable for different tasks, the gripper is able to achieve a wide clamping range and a superior adaptability for objects in different size. It would be less costly for manipulating multi-targets with this type of gripper compare to those grippers limited in a determinate range. In addition, the end effectors of three fingers can be interchangeable in shape, material, and size according to different shaped manipulating targets. Moreover, the third finger aided for releasing changed the acceleration of the releasing object by knocking on the object. The releasing process aided with a third finger overcomes the adhesion force between the third finger and the objects, which achieves an effective release for objects. In Section 1, we have given a general talk about some previous research on micro-grippers. The detailed structure and motion analysis of the proposed gripper are described in Section 2. Simulations and Calibration of the gripper are presented in Section 3. Experimental results of pick-and-place micro-objects by double-finger or triple-finger gripper and discussions are included in Section 4.

## 2. Design and Motion Analysis of Gripper

**Figure 1:**
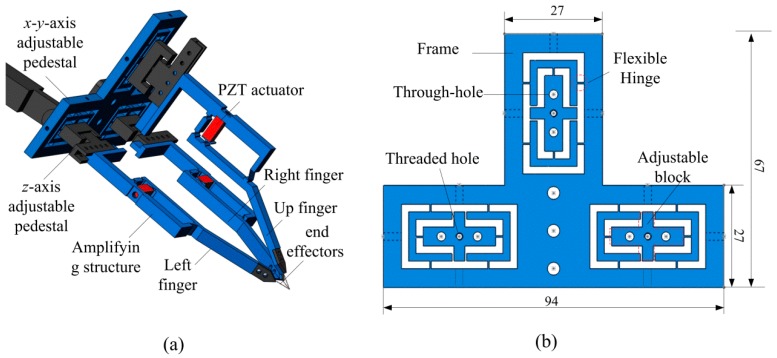
(**a**) Schematic drawing of the triple-finger gripper; (**b**) Front view of the *x*-*y* adjustable pedestal.

Figure 1a shows a schematic diagram of the micro-gripper with a triple-finger. The overall size of the gripper is about 107 mm × 94 mm × 67 mm. The left, right, and the up fingers are assembled in an *x*-*y*-axis adjustable pedestal via three adjustable blocks. Each finger contains a *z*-axis adjustable pedestal, an amplifying structure, and an end-effector. The amplifying structure is driven by a piezoceramic (PZT) actuator which can generate a maximum displacement of 10 μm. The structure of the *z*-axis adjustable pedestal is specially designed with five pairs of thread holes on the both sides of the groove to adjust the initial clamping range of the gripper. By fixing the fingers on the different holes, the gripper can reach a wide clamping range to meet the requirements of micro-objects with different sizes and shapes. According to different manipulating micro-objects, variable end effectors can be replaced, such as tungsten tipped probes, fibers, atomic force microscope (AFM, Olympus, Tokyo, Japan) probes and so on.

**Figure 2:**
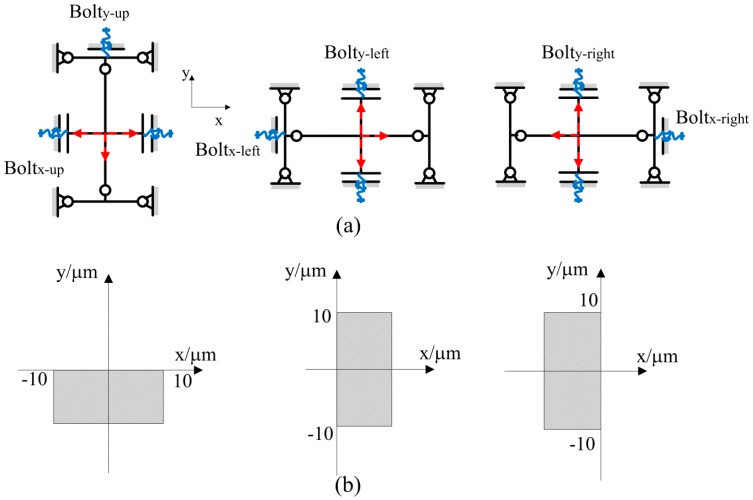
(**a**) Motion diagram of the *x*-*y* adjustable pedestal; (**b**) The displacements of the up, left, and right blocks in *x*-*y* plane, respectively.

The *x*-*y*-axis and *z*-axis adjustable pedestals are employed to expand the range of the clamping objects and avoid deviation in a certain range. Figure 1b shows a front view of the *x*-*y*-axis adjustable pedestal. It contains three adjustable blocks. Two through-holes and one threaded-hole in each adjustable block are used to connect and modulate the *z*-axis adjustable pedestal. Flexible hinges are designed in between the adjustable blocks and the frame of the pedestal. By using swivel bolts through the adjustable holes in the frame, every block is able to make micrometric displacements along *x*- and *y*-axis. Figure 2a shows a simplified diagram of the *x*-*y*-axis motion of the blocks. The frame is assumed as the fixed end and the flexible hinges are simplified as cross lines. Every adjustable block is driven by three swivel bolts along *x* and *y*-directions as identified in red arrow.

Taking the center of every adjustable block as the datum point, the displacement range of every block is calculated. As shown in Figure 2b, the movement range of the up block is from −10 to 10 μm in *x*-axis and from −10 to 0 μm in *y*-axis. The displacement range of the left block is from 0 to 10 μm in *x*-axis and from −10 to 10 μm in *y*-axis. The range of the right block is from −10 to 0 μm in *x*-axis and from −10 to 10 μm in *y*-axis. With these adjustable blocks, three separate fingers can be aligned accurately. With a resolution of micrometers scale, the gripper can be assembled in a relatively high precision for clamping micro-objects.

**Figure 3:**
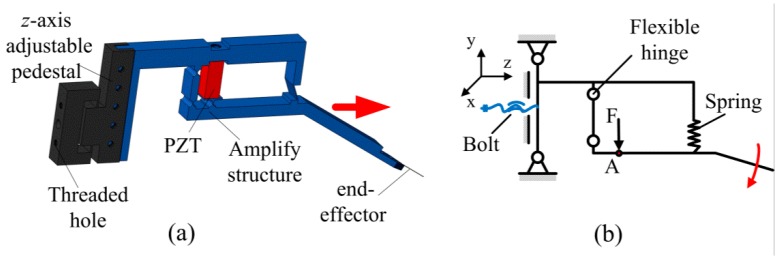
Assembled single finger structure and motion diagram of a single finger.

Figure 3a shows the assembled single finger structure and Figure 3b shows the simplified motion diagram of a single finger. The bolt can adjust the displacement of the finger along *z*-axis direction in a certain range. The amplification mechanism is essentially a micro-leverage structure, which contains two flexible hinges, a spring structure, and an embedded PZT actuator. The two flexible hinges can be considered as a fulcrum point. The PZT actuator is pre-tightened initially by a bolt and it produces a driving force F on the amplfying structure at point A. The spring structure helps the finger to recover the original position as the driving force of the PZT transducer is removed.

**Figure 4:**
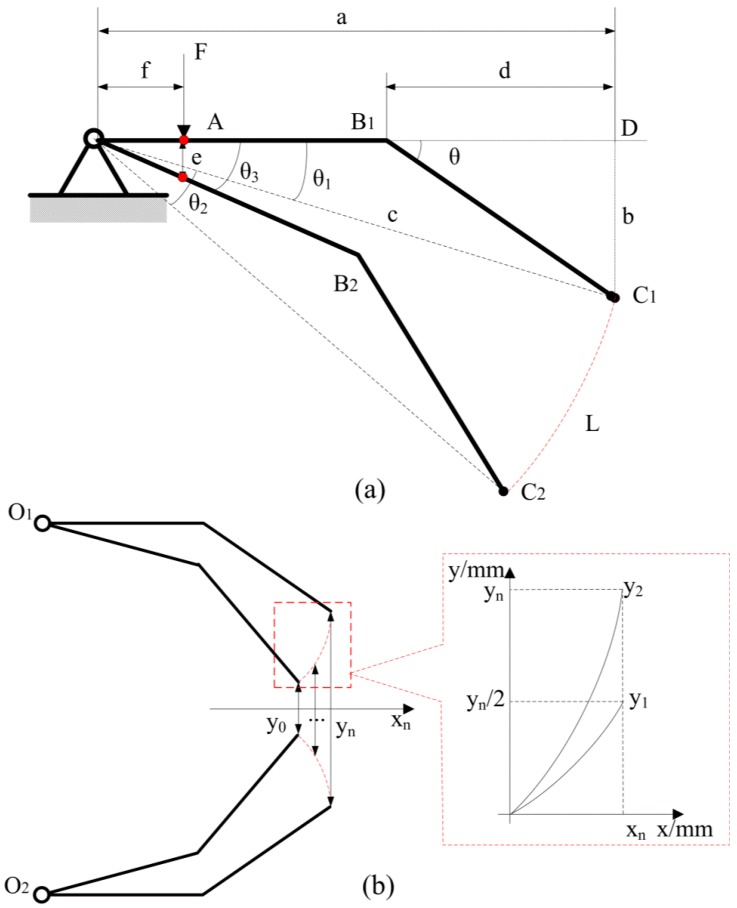
(**a**) Simplified diagram and the maximum output of a single finger (unit: mm); (**b**) Trajectories of left and right fingers and gripping distance between these two fingers.

Figure 4a shows the dimension parameters of a simplified single finger. θ_1_ is the angle between OB_1_ and OC_1_. *a* is the length of OD, i.e., 62.5 mm, and *d* is the length of B_1_D, i.e., 30 mm. *c* is the length of OC_1_.

Assuming the PZT actuator applied a driving force *F* at point A, the output displacement of point A on the finger is *e*. The moving angle can be expressed as:

(1) $$\tan\theta_{3}=e/F$$

The maximum output displacement of piezoelectric transducer is 0.01 mm, which is the length of *e*. θ_2_ is the moving angle of the finger, which equals to θ_3_. Trajectory length *L* of the single finger is calculated to be 0.118 mm.

Figure 4b shows the trajectory between the left and right fingers. The motion of fingers is similar to a lever turning around a fixed point. Distances between the endings of finger can represent the range of gripping targets. If *y*_0_ is zero, gripping distance between left and right fingers is shown in Figure 4b. These values of distance can be represented using functions as follows. The variable named *y*_1_ represents distances between the finger’s end and the gripper’s center line, which can be expressed as:

(2) $${\left[x_{1}+c\times \cos\left(\theta_{1}+\theta_{2}\right)\right]}^{2}+{\left[y_{1}-c\times \sin\left(\theta_{1}+\theta_{2}\right)\right]}^{2}=c^{2},\left(0\leq x\leq x_{n}\right)$$

where *b* is 17.33 mm, *a* is 62.5 mm, and *c* is 64.86 mm. Therefore, *x_n_* can be calculated as:

(3) $$x_{n}=c\times \sin\left(\theta_{1}+\theta_{2}\right)-b$$

The variable named *y*_2_ represents distances between the left finger’s end and the right’s one which can be expressed as:

$${\left[x_{1}+c\times \cos\left(\theta_{1}+\theta_{2}\right)\right]}^{2}+{\left[\frac{y_{2}}{2}-c\times \sin\left(\theta_{1}+\theta_{2}\right)\right]}^{2}=c^{2},\;\left(0\leq x\leq x_{n}\right)$$

## 3. Simulation and Calibration of Gripper

**Figure 5:**
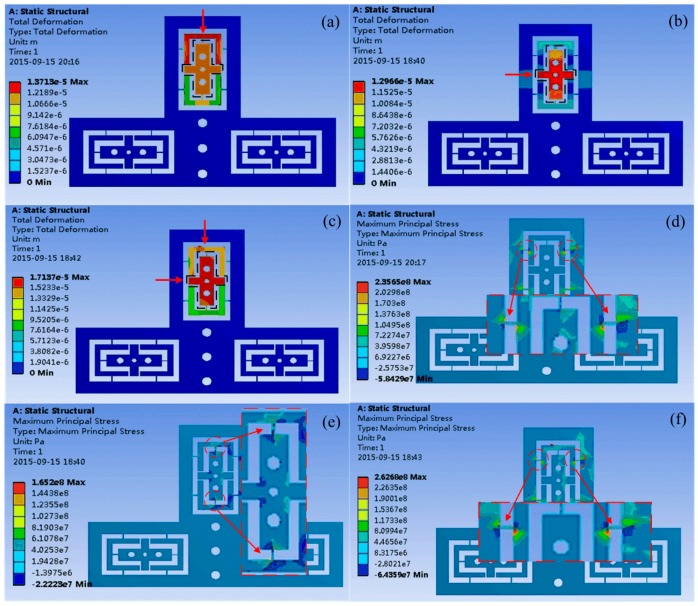
Deformation analysis of the *x*-*y*-axis adjustable pedestal as the applied force of bolt is in (**a**) *y*-axis, (**b**) *x*-axis, and (**c**) both *x*- and *y*-axis. Stress analysis of the *x*-*y*-axis adjustable pedestal as the applied force of blot is in (**d**) *y*-axis, (**e**) *x*-axis, and (**f**) both *x*- and *y*-axis.

The force-displacement and yield limit of the *x*-*y*-axis adjustable pedestal are analyzed by using finite element analysis (FEA) software. The spring steel with Young’s modulus of 200 GPa is selected as the pedestal material. In the simulation, the swivel bolt applies a concentrated force of 50 N on top adjustable block in three strategies. In Figure 5a, the applied force is in negative direction of *y*-axis, and the maximum displacement is obtained as 13.7 μm. Similarly, as the force is applied in positive direction of *x*-axis, the maximum displacement is 13.0 μm, as shown in Figure 5b. In Figure 5c, a maximum displacement of 17.1 μm can be obtained when a combined force along negative *y*-direction and positive *x*-direction is applied on the top block. For these three cases, the displacement of the block is in a uniform distribution. Figure 5d–f shows the corresponding stress distribution of these three cases. It is found that the stress concentration occurs at the flexible hinges. The maximum stress of the hinges is about 262 MPa, which is far less than its yield strength of 520 MPa. It is verified that the proposed design meets the application requirements.

**Figure 6:**
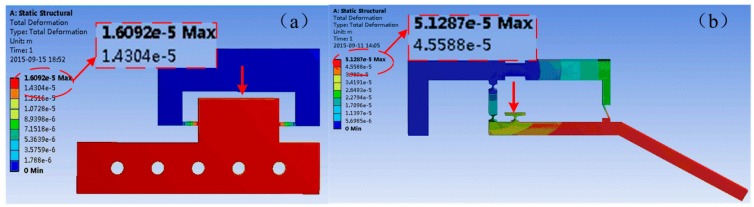
(**a**) Deformation analysis of the *z*-axis adjustable pedestal as the applied force of bolt is in *z*-axis. (**b**) Deformation analysis of a single finger as the driving force of PZT transducer is applied.

A similar deformation analysis of the *z*-axis adjustable pedestal and amplifying structure are conducted and shown in Figure 6. As a concentrated force of 50 N is applied, a displacement of 16.1 μm can be achieved along *z*-axis as seen in Figure 6a. In Figure 6b, a maximum deformation of 51.3 μm is obtained at the driving force of PZT transducer of 50 N.

**Figure 7:**
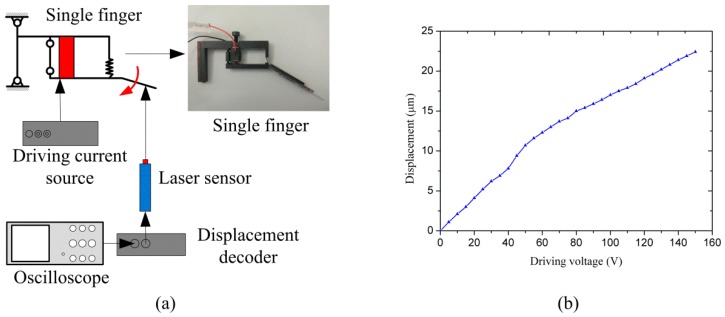
(**a**) Displacement calibration testing system; (**b**) Output displacement of single finger.

Figure 7a shows the displacement calibration testing system. It consists of a power driving source, a single finger, a laser sensor, a displacement decoder, and an oscilloscope. When a driving voltage is applied from 0 to 150 V, the PZT transducer can monotonously generate a displacement output from 0 to 10 μm. The end tip displacement of the single finger against driving voltage can be measured by the laser sensor and recorded by the oscilloscope via a displacement decoder. As seen in Figure 7b, the actuation calibration curve is nonlinear, which is due to the arc trajectory of the finger-tip. Besides, errors in machining and assembly process may also have an impact on the output displacement. The result shows that the displacement range of the fingertip is from 0 to 21.7 um as the driving voltage varying from 0 to 150 V. Therefore, the clamping range of the left and right fingers is 43.4 μm. The resolution of the output displacement is 145 nm/V.

## 4. Experiments and Discussion

**Figure 8:**
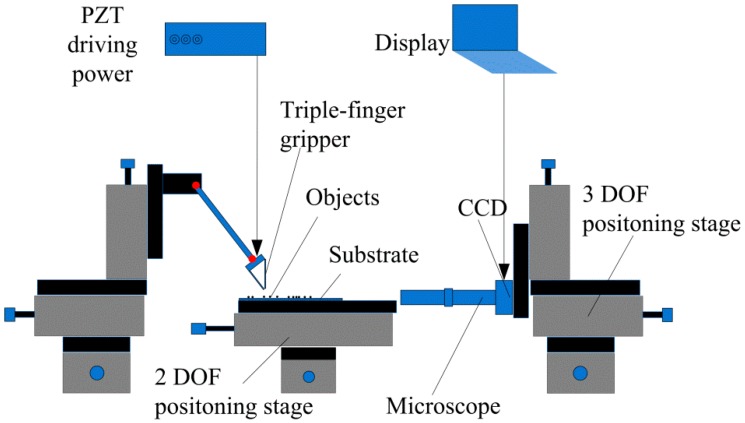
The manipulation setup of the triple-finger gripper.

As shown in Figure 8, a manipulation system is constructed to demonstrate the capability of the triple-finger gripper. The gripper is installed on a 3-DOF positioning stage (Suruga Seiki, Shisioka, Japan), by which the gripper can be adjusted to the target location. The objects and substrate are placed on a 2-DOF positioning stage, which assist the gripper to locate the objects. A microscope fixed on the other 3-DOF positioning stage helps to identify the objects and get clear images. Three fingers of the gripper are controlled by three piezoelectric ceramics via three channels. A charge-coupled device (CCD) camera (Daheng Imavision, Beijing, China) is used to record the manipulation processing in real time. Several kinds of end effectors are fixed on the gripper to manipulate various objects.

### 4.1. Experiment of Double-Finger Gripper

**Figure 9:**
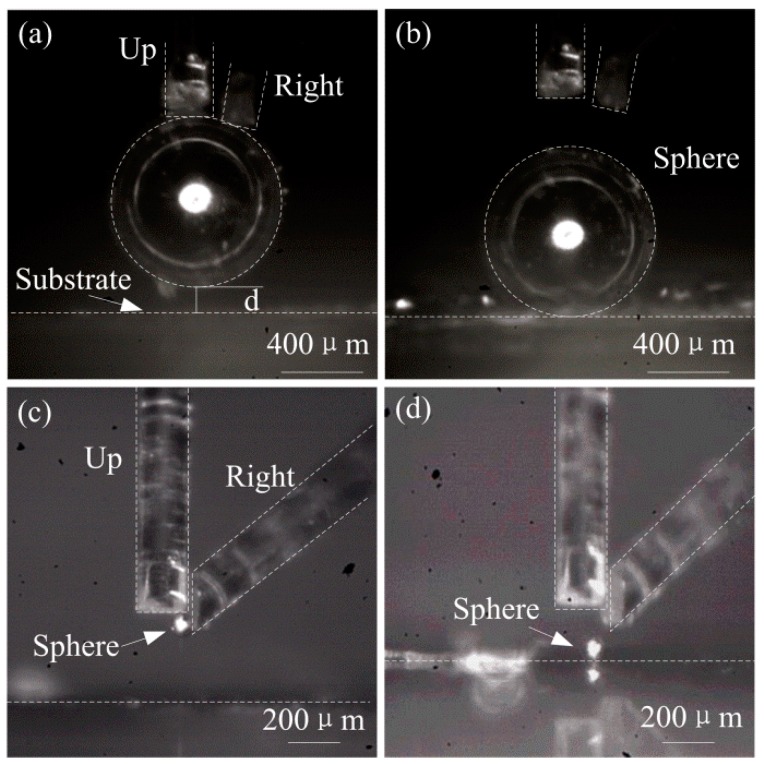
(**a**) Pick and (**b**) place a glass hollow sphere with a diameter of 800 μm; (**c**) Pick and (**d**) place an iron sphere with a diameter of 80 μm.

The manipulation experiment of double-finger gripper is firstly conducted. The processes of picking and placing a glass hollow sphere with a diameter of 800 μm and an iron sphere with a diameter of 80 μm are illustrated in Figure 9. Two optical fibers are used as the up and right end effectors of the fingers. Due to the adhesion force, the up and right fingers can pick up the sphere on top of the substrate with a distance of d as shown in Figure 9a. Since the up finger is controlled by the driving PZT transducer, it can provide a vertical vibration to release the adhered sphere as shown in Figure 9b. Similarly, Figure 9c,d show the process of picking and placing an iron sphere with a small diameter of 80 μm. In the experiment, the manipulating objects are different, and the diameter of the objects varies from 80 to 800 μm. It is seen that the gripper is adaptable for multi-target micromanipulation with a quite large clamping range by the adjustable pedestal and PZT driving amplifier structure introducing for each finger.

### 4.2. Experiment of Triple-Finger Gripper

**Figure 10:**
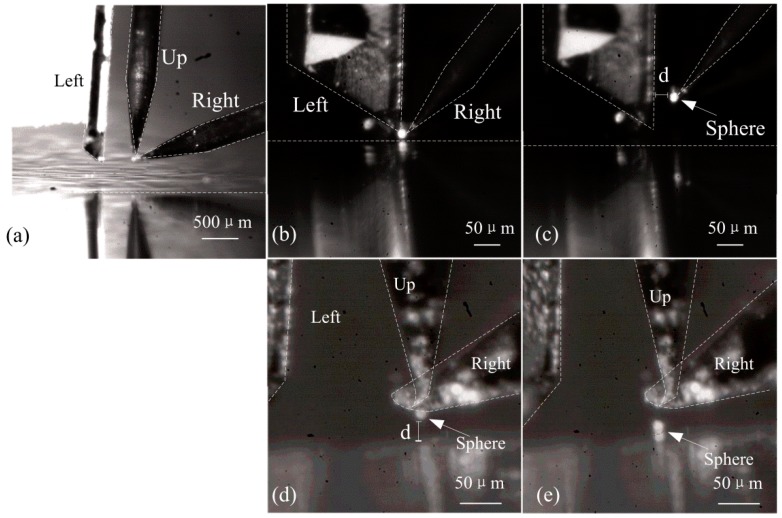
(**a**) Triple-finger gripper; (**b**) Gripping the boron silicate sphere; (**c**) Sphere failed to release from the fingers; (**d**) Fingers with adhered sphere; (**e**) Releasing process with the up finger.

Figure 10a shows the triple-finger gripper including left, right, and up end effectors on three fingers. The left end effector is an AFM probe. The up and right end effectors are two tungsten tipped probes. The triple-finger gripper is able to clamp boron silicate sphere with a diameter of 10 μm in *y*-direction as seen in Figure 10b. However, without the assist of the third up finger, the sphere failed to release from the fingers due to the adhesion force. It can be seen from Figure 10c that, as the left and right finger open, the sphere is adhered to one of the fingers.

To release the adhered sphere, the up finger is adjusted to the position that above the sphere in Figure 10d. By applying a driving voltage of 20 V and a sinusoidal signal to the PZT transducer, the up finger is able to generate a high frequency vibration, and the sphere is able to release from the right finger easily. Figure 10e shows the enlarged image after releasing process. It is verified that a triple-finger gripper with PZT actuation mechanism gains the advantage to overcome the adhesive force in micro-manipulating process. Therefore, it has a large manipulating range for various micro-objects.

## 5. Conclusions

This paper demonstrates a PZT actuated gripper which has the ability to pick and release multi-sized objects. The gripper contains three separated fingers, which can be changed in manipulating range through adjustable blocks. The gripper can be fabricated by low-cost machining methods. The three fingers can be assembled with variable end effectors such as tungsten probes, fibers, or AFM probes. The output resolution and displacement range of each single finger is as high as 145 nm/V and 21.7 μm, respectively. To release the objects, the up finger can be controlled to provide vertical vibration. In the experiment, the glass hollow sphere with a diameter of 800 μm and the iron sphere with a diameter of 80 μm were picked and placed by the up and right fingers. The iron sphere of 10 μm in diameter was gripped by the tungsten tipped probes and released by the vibration of the up finger. All these results showed that the triple-finger gripper works well for multi-target micromanipulation.

Future work will focus on force control of the gripping process and optimization of adjustable mechanism. Force control of the griper would improve the efficiency and avoid destruction in manipulation. Optimization of adjustable mechanism would raise the accuracy of gripper and manipulate smaller objects. Additionally, more manipulating strategies will be investigated to pick, transport, and place more targets in the future, which will provide experimental guidance for the design of special micro-manipulation tools. Applications of this devised micro-gripper in super-resolution imaging and inertial confinement fusion adhesion problem solving will be further studied in the future.
